# Radiology of fibrosis part III: genitourinary system

**DOI:** 10.1186/s12967-024-05333-1

**Published:** 2024-07-03

**Authors:** Sofia Maria Tarchi, Mary Salvatore, Philip Lichtenstein, Thillai Sekar, Kathleen Capaccione, Lyndon Luk, Hiram Shaish, Jasnit Makkar, Elise Desperito, Jay Leb, Benjamin Navot, Jonathan Goldstein, Sherelle Laifer, Volkan Beylergil, Hong Ma, Sachin Jambawalikar, Dwight Aberle, Belinda D’Souza, Stuart Bentley-Hibbert, Monica Pernia Marin

**Affiliations:** 1 Department of Biomedical Sciences, Humanitas University, Milan, Italy; 2 Department of Radiology, Columbia University Irving Medical Center, 630 W 168th Street, New York, NY 10032 USA

**Keywords:** Fibrosis, Thoracic organs, Imaging

## Abstract

Fibrosis is a pathological process involving the abnormal deposition of connective tissue, resulting from improper tissue repair in response to sustained injury caused by hypoxia, infection, or physical damage. It can impact any organ, leading to their dysfunction and eventual failure. Additionally, tissue fibrosis plays an important role in carcinogenesis and the progression of cancer.

Early and accurate diagnosis of organ fibrosis, coupled with regular surveillance, is essential for timely disease-modifying interventions, ultimately reducing mortality and enhancing quality of life. While extensive research has already been carried out on the topics of aberrant wound healing and fibrogenesis, we lack a thorough understanding of how their relationship reveals itself through modern imaging techniques.

This paper focuses on fibrosis of the genito-urinary system, detailing relevant imaging technologies used for its detection and exploring future directions.

**Supplementary Information:**

The online version contains supplementary material available at 10.1186/s12967-024-05333-1.

## Background

This is the third instalment of a three-part series regarding the radiology of fibrosis across organs. This installment concerns genitourinary organs, in particular, the kidneys, the bladder, and the prostate. The prior parts of this series are titled “Radiology of Fibrosis Part I: Thoracic Organs” and “Radiology of Fibrosis Part II: Abdominal Organs”. By structuring our work in this manner, we hope to have provided the readership with a clear image of a complex issue, paving the way for future betterment of clinical practice.

As discussed in the first third of this work, fibrosis is a pathological process characterized by abnormal deposition of connective tissue and improper tissue repair in response to sustained injury [1]. It can impact any organ, leading to severe structural and functional dysfunction and even failure [2, 3]. Data suggests fibrosis may account for the insurgence of up to 20% of all cancers and for up to 45% of deaths in industrialized nations, thus emphasizing the relevance and importance of pursuing a more thorough understanding of wound healing, chronic inflammation, and fibrosis [2, 4].

The physiological wound healing process involves the processes of hemostasis, inflammation, proliferation, and remodeling [5, 7–14]. Aberrant tissue repair, instead, determines the development of chronic inflammation, excessive fibroblast proliferation, heightened collagen deposition, and, ultimately, an imbalanced alternation of scar formation and remodeling [3, 5].

While extensive research has already been carried out on the topics of aberrant wound healing and fibrogenesis, we lack a thorough understanding of how their relationship reveals itself through modern imaging techniques. Considering the profound implications that advancements in this field may carry, and with the objective of exploring and expanding upon our current understanding, this study seeks to study fibrosis across various organs of the genitourinary system and catalog the foremost imaging technologies utilized for its identification.

## Renal fibrosis

### Mechanism of injury

Renal fibrosis is an unfortunate sequel of extensive, ongoing tissue damage and is the final phase common to most pathological kidney repair processes [15, 16]. It is characterized by deposition of extracellular matrix (ECM) which can affect all compartments of the renal parenchyma leading to organ failure [15]. Resident fibroblasts are of vital importance for the induction and advancement of fibrotic disease [17]. In response to injury, they differentiate into myofibroblasts acquiring the capacity to produce large amounts of ECM and displaying a more pro-inflammatory phenotype [17]. Soon after initial insult to the kidney parenchyma, resident fibroblasts activate the nuclear factor κB (NF-κB) signaling pathway leading to the production of pro-inflammatory cytokines which advance inflammation [17]. This stage of the wound healing process has been found to be potentially reversible through the administration of anti-inflammatory agents [17]. The same nuclear factor, NF-κB, has been linked to an increase in the activity of sodium hydrogen exchanger 3, a major proximal tubule transporter responsible for sodium reabsorption and albumin endocytosis [17, 18]. Increased plasma albumin, increases oncotic pressure and reduces albumin gradient producing increased filtration of intravascular fluid into the interstitium, leading to inflammation and edema [19].

## US

**Fig. 1:**
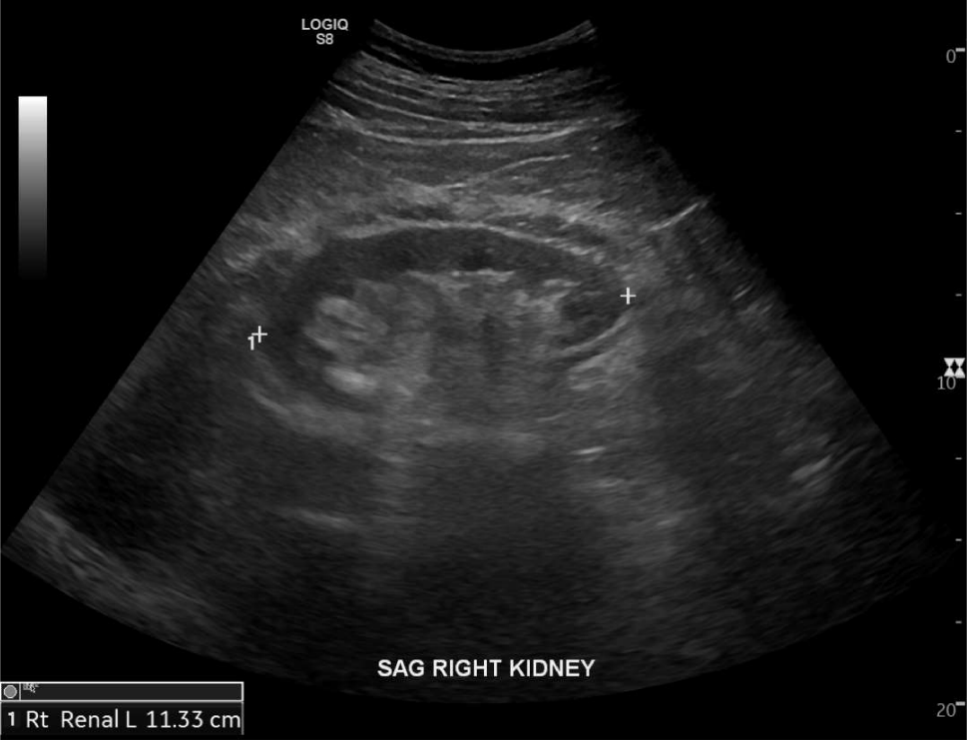
Ultrasound showing diffuse cortical thinning and mildly echogenic renal parenchyma

Conventional renal US is often used in the initial evaluation because it is safe, easy and inexpensive to perform [20]. Renal US features, such as increased parenchymal echogenicity and decreased renal size and parenchymal thickness can be easily assessed [20] (Fig. 1). For this reason, parenchymal echogenicity is a commonly used marker for nephropathy [20]. However, this marker is subjective, not quantitative and often fails to detect renal abnormality [20]. Thus, conventional renal US is generally uninformative in evaluating the progression of chronic kidney disease (CKD) [20]. For this reason, a superior alternative to this imaging modality has been offered in the form of ultrasound elastography (USE) [20, 21]. USE is a low-cost and non-invasive US imaging technique for the assessment of tissue stiffness based on the pathological and physiological principle that fibrotic tissues have different elasticities compared to normal tissues [20, 22, 23]. The same working principle applied in magnetic resonace elastography (MRE) [21]. Two main USE subtypes are employed today: strain elastography (SE) and shear wave elastography (SWE). Thus far, most research has been based on SWE [23]. In SWE, real-time rapid tissue deformation induced by an external compression device generates a shear wave that propagates perpendicularly to the main US beam [20, 23, 24]. The US scanner can monitor the tissue displacement, measuring the time-to-peak displacement and the recovery time in order to quantify physical strain within the tissue [20, 24]. Preliminary results are promising, showing renal elasticity to be consistently associated with renal deterioration in patients with CKD [23, 24]. This technology has the potential to help assess early alterations in renal function and pathological alterations, thus calling for further investigation into the in vivo utility of USE [21, 23]. However, previous studies that investigated the relationship between the SWE and renal function and fibrosis are not consistent with each other finding both significant positive and negative correlations [20, 21, 23]. To explain this discrepancy, we must consider the fact that kidney stiffness is not only related to fibrosis, indeed there are several additional influencing factors [22, 23]. For example, the heterogeneity of patient cohorts, patient age, variations in renal blood flow, the presence of atherosclerosis, coronary artery disease, hypertension, hydronephrosis, degree of bladder distension, and the wide array of underlying cause (diabetic nephropathy, nephrosclerosis, glomerulonephritis, etc.) [20, 22, 23]. The lack of consensus on the elastic changes in CKD means the results of USE should be interpreted carefully and studies should be furthered [20, 23]. An example of renal US showing mildly echogenic renal parenchyma is provided in Fig. 1. Finally, non-US technologies dedicated to the detection of renal fibrosis are MRI, DWI MRI, MRE, ASL fMRI, BOLD MRI, and PET [21]. Contrast enhanced CT is not often prescribed in nephrological patients due to the high risk of contrast induced acute kidney injury (AKI) [26, 27].

## MRI

**Fig. 2:**
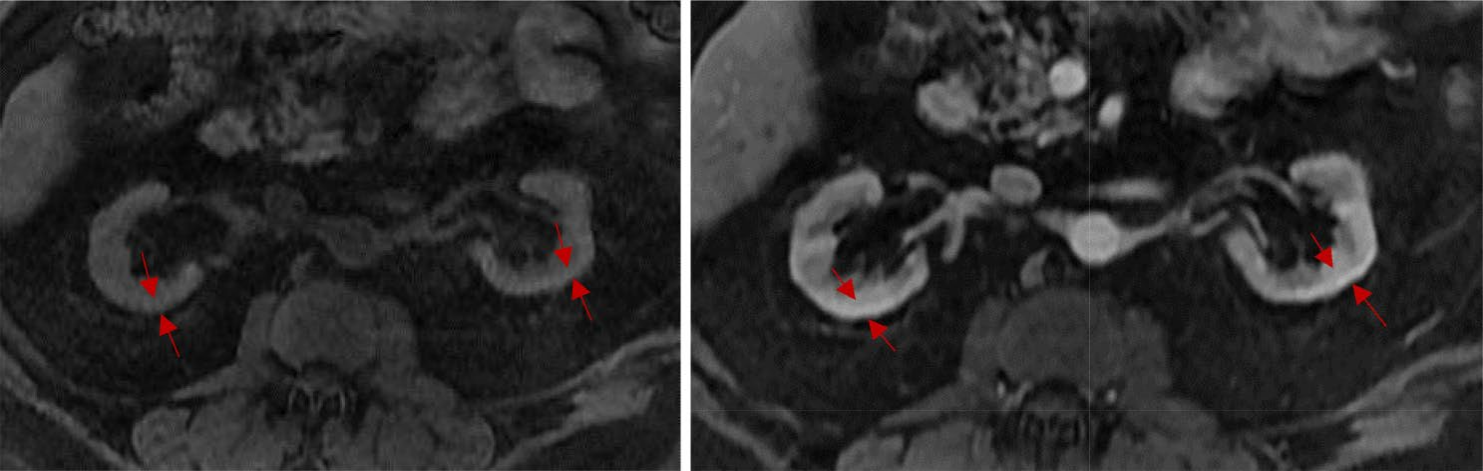
Renal MRI. Pre and post T1 show homogenous, symmetric renal cortical thinning (red arrows)

**Fig. 3:**
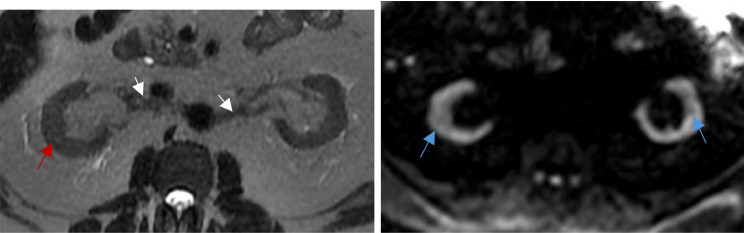
Axial T2 DWI-MRI with mild homogeneous T2 hypointensity (red arrow), no fluid in the renal collective systems (white arrows), and diffuse cortical restricted diffusion (blue arrows)

The gold standard non-invasive renal fibrosis diagnostic technique is represented by MRI (Fig. 2). In particular, diffusion weighted magnetic resonance imaging (DWI) (Fig. 3) and magnetic resonance elastography (MRE) [25]. Other newer MR-based technologies are arterial spin labeling functional MRI (ASL fMRI), functional MRI, and blood oxygenation-level-dependent (BOLD) MRI [21].

DWI, widely recognized as a powerful tool for imaging renal microstructure and function, is a non-invasive imaging technique that utilizes in vivo water molecule diffusion mapping to generate contrast in MR images [21, 25]. Water motion in biological tissues is restricted by tissue components such as fibers, macromolecules, and cell membranes [21]. Thus, the deposition and accumulation of ECM in fibrotic kidneys as well as tubular atrophy influences water diffusion patterns [21]. The commonly used parameter to quantify diffusion differences from the physical diffusion coefficient is called the apparent diffusion coefficient (ADC) value which has been found to be considerably and consistently lower in patients with CKD than in healthy controls [21, 25]. The acquired images have low spatial resolution and are subject to distortions caused by respiratory motion and water protons present at the bowel-tissue interface [21, 25]. Recently, a new technique termed Readout Segmentation Of Long Variable Echo-trains (RESOLVE) has been developed to reduce DWIs susceptibility to these artifacts [21, 25]. Further technical developments are needed to validate its in vivo applicability [21, 25]. An example of DWI-MRI is provided in Fig. 3 and shows mild homogeneous T2 hypointensity, no fluid in the renal collective systems, and diffuse cortical restricted diffusion.

The accumulation of ECM during development of renal fibrosis typically augments tissue stiffness, which may, thus, serve as a good biomarker of renal fibrosis [21, 28]. MRE is a novel MRI-based technique that directly and noninvasively measures tissue stiffness by studying the propagation of vibrational acoustic shear waves in the tissue [21, 25, 28, 29]. This technology promises early detection, staging, and prevention of renal disease while reducing the need for bioptic procedures and eliminating inherent sampling errors [28, 30]. While initial results are encouraging, further studies are needed to validate the in vivo utility of MRE [25, 28, 30]. Heterogeneous tissue texture and changes in renal perfusion pressure have been found to modulate the measurement of parenchymal stiffness, often masking the presence of fibrosis [21, 28, 29, 31]. Patients with CKD and renal edema have been found to present with frequently reduced renal blood flow (RBF), an early marker of renal damage, and consequently decreased tissue turgor [31–34]. In turn, decreased tissue turgor has been found to determine a counterintuitive decrease in fibrotic kidney MRE measurements [32].

Based on this knowledge, a pilot study lead by Brown et al. paired MRE technology to arterial spin labeling (ASL), a noninvasive fMRI technique through which RBF can be reliably quantified and which promises early-stage detection of CKD [32–34]. In ASL, water in arterial blood is used as an endogenous tracer to map of regional perfusion [35, 36]. Two image types are captured: a contrast-free control image and a labeled image in which an electromagnetic pulse magnetizes arterial blood [36]. The magnetized image is subtracted from the control image to generate a map of RBF in which signal intensity is proportional to perfusion [36]. Wherever renal perfusion values are found to be significantly lower or lacking, edema and/or fibrosis can be suspected [31, 32, 34]. Renal perfusion by ASL has been validated against reference methods and has good reproducibility [36]. Its short acquisition time enabled combination with other MRI techniques like MRE [33, 36]. Furthermore, no ionizing radiation or nephrotoxic contrast agents such as gadolinium, are required [35, 36]. Instead, a key challenge for ASL fMRI is the inherently limited signal-to-noise ratio necessitating repeated measurements to allow data averaging, thus, even though feasibility has been demonstrated in the kidneys, its clinical utility is still under investigation [31, 33]. In Brown et al.’s study, the combination of ASL and MRE provided a useful tool and yielded promising results [32].

The degree of inflammation and edema at the level of single renal compartments is appreciable via fMRI with voxel-wise mapping of longitudinal (T1) and transverse (T2) relaxation times [37, 38]. This method of tissue characterization is based on timing the process of spinning protons’ alignment and loss of alignment with the external magnetic field following an electromagnetic pulse. On T2 weighted images, the molecular imaging probe superparamagnetic iron oxide (SPIO) appears darkened indicating reduced relaxation time [39]. SPIO is ingested by macrophages and is thereby accumulated in inflamed tissues [34]. The resulting images can therefore serve as proxy for the detection of macrophage infiltration, revealing the extent of inflammation. In the absence of SPIO, wherever T2 relaxation time is found to increase, an increase in tissue water content is to be expected allowing for edema detection [21, 38]. Despite these promising findings, the sensitivity and specificity of T1 and T2 mapping are low and their ability to detect renal fibrosis in vivo remains to be investigated [21].

Under healthy conditions, blood and tissue oxygenation are at equilibrium [21]. Instead, in fibrotic kidneys, glomerular injury promotes microvascular obliteration, limiting the access of deoxyhemoglobin to the tissue, and leading to hypoperfusion and chronic hypoxia, recognized as the final common pathway to end-stage renal failure [21, 40, 41]. BOLD MRI offers an indirect and noninvasive method with which to detect the presence of intravascular deoxyhemoglobin in kidney interstitium, without the need for contrast agents [21, 40–42]. This technology exploits the fact that magnetic properties of hemoglobin depend on its oxygenated state: the higher local deoxyhemoglobin, the higher tissue’s T2* relaxation time, and the lower local tissue oxygen content [21, 41]. In so doing, regional renal oxygenation may be taken as an endogenous marker of renal fibrosis [21, 40].

## Nuclear medicine

It is possible to single out regions of kidney inflammation with PET imaging technology as well. PET provides a quantitative measure of inflammatory markers such as chemokine receptor 4 (CXCR4), a transmembrane receptor involved in the transit of white blood cells to wound site during inflammation can be labelled and imaged with 68Ga-pentixafor [34, 43–45]. CXCR4 targeted PET enables dependable and precise detection of leukocyte infiltration into renal tissue thus allowing for the detection of inflammation [34, 43, 46].

## Future directions

**Table 1:** Renal fibrosis imaging – pros/cons with respects to the gold standard

Breast Fibrosis Imaging	US	PROs	Non-invasive, Easy, Low-cost, Readily available, Well tolerated, No ionizing radiation
		CONs	Operator dependent, Not quantitative, Generally uninformative in evaluating CKD progression, Dependent on patient habitus
	USE-SE/SWE	PROs	Low-cost, Non-invasive, Early diagnosis, Readily available, Well tolerated, Quantitative, No ionizing radiation
		CONs	Operator dependent, Stiffness depends not only on fibrosis but also on patient age, variations in renal blood flow, the presence of atherosclerosis, coronary artery disease, hypertension, hydronephrosis, degree of bladder distension, and the wide array of underlying cause, Dependent on patient habitus
	DWI MRI^1^	PROs	Non-invasive, No contrast agent, No ionizing radiation
		CONs	Low spatial resolution, Sensitive to patient movement/respiration, High cost
	MRE	PROs	Non-invasive, Direct measurements, early detection, reduces the need for biopsies and their inherent sampling error, No ionizing radiation
		CONs	Stiffness depends not only on fibrosis but also on patient age, variations in renal blood flow, the presence of atherosclerosis, coronary artery disease, hypertension, hydronephrosis, degree of bladder distension, and the wide array of underlying cause, High cost
	ASL fMRI^2^	PROs	Non-invasive, Quantitative, Early detection, No contrast agent, Good reproducibility, Low acquisition time, No ionizing radiation
		CONs	Low signal-to-noise ratio, High cost
	fMRI	PROs	Non-invasive, No ionizing radiation
		CONs	Requires contrast medium, Low sensitivity/specificity, High cost
	BOLD MRI	PROs	Non-invasive, No contrast agents, No ionizing radiation
		CONs	Indirect, High cost
	PET-CT	PROs	Quantitative
		CONs	Use of contrast agent, Use of ionizing radiation, High acquisition time, Low availability, High cost

^1^Gold standard, ^2^Promising future techniques

Benefits and drawbacks of each imaging technique discussed above are summarized in Table 1. Among the proposed alternatives, the authors of this review feel ASL adjunct MRE to be the most promising. The combination of these noninvasive MRI-based imaging techniques allows for the quantification of renal fibrosis while accounting for reduced RBF’s masking effect [21, 25, 28, 29, 32–34]. MRE is a novel MRI-based technique that directly and noninvasively measures tissue stiffness by studying the propagation of vibrational acoustic shear waves in the tissue [21, 25, 28, 29]. This technology promises early detection, staging, and prevention of renal disease while reducing the need for bioptic procedures and eliminating inherent sampling errors [28, 30]. Even so, changes in renal perfusion pressure have been found to modulate the measurement of parenchymal stiffness, often masking the presence of fibrosis [21, 28, 29, 31]. Patients with CKD have been found to present with frequently reduced RBF, an early marker of renal damage, and consequently decreased tissue turgor which determines a counterintuitive decrease in fibrotic kidney MRE measurements [31–34]. Thus, ASL, a noninvasive fMRI technique, has been implemented to reliably quantify RBF, promising to improve early-stage detection of CKD [32–34]. This technology uses water in arterial blood as an endogenous tracer with which to map regional perfusion [35, 36]. ASL RBF measurements have been validated against reference methods and have good reproducibility [36]. Its short acquisition time enables combination with other MRI techniques like MRE [33, 36]. Furthermore, no ionizing radiation nor nephrotoxic contrast agents are required [35, 36].

## Bladder fibrosis

### Mechanism of injury

Interstitial cystitis (IC) is a severe inflammation of the urinary bladder with mucous membrane destruction which can provoke bladder fibrosis [47, 48]. Bladder pain syndrome (BPS) is caused by chronic inflammation associated with debilitating bladder storage symptoms such as urgency, frequency, and nocturia [47, 48]. Biopsy of affected bladder walls reveals the presence of chronic inflammation in the form of high T and B cell expression, high mast cell density, immune cell aggregation, and IL-6, IL-10, and IL-17 A overexpression [47]. IC corresponds to the inflammatory phase of bladder disease, having the potential for the development of bladder fibrosis with upregulation of collagen and fibronectin production, excessive deposition of ECM within the lamina propria, synthesis of myofibroblasts, and decrease in capillary density [47]. The condition’s inflammatory nature means treatment with anti-inflammatory medication has been found to limit IC/BPS signs and symptoms while restoring physiological anatomy and functionality of the urinary bladder [49].

### Cystoscopy

In clinical settings, suspected ICs should be mandatorily investigated via cystoscopy, a planar imaging technique which allows for direct, real-time imaging of the bladder wall [47, 50, 51]. The gold standard diagnostic practice consists of symptom evaluation, cystoscopic findings (Hunner’s lesions and bleeding), and exclusion of alternative disorders, such as bladder carcinoma, endometriosis, infection, and bladder stones [48, 50, 51]. During cystoscopy the bladder is distended to full capacity and then drained [50]. Continuous (pre/intra/post dilation) inspection of the luminal surface of the bladder wall is carried out with the aim of identifying Hunner’s ulcers, erythematous mucosal patches with small vessels radiating toward a central scar [47, 50, 51]. Rigid cystoscopes are equipped with large forceps to facilitate the sampling of bladder biopsies at roughly half full bladder capacity [50]. While IC presents with no pathognomonic histology, bioptic procedures may reveal typical findings, such as a denuded epithelium, ulceration, chronic inflammation, and raised mast cell count [48]. Even so, the technique’s small field of view (FOV) only allows for gross morphological discrimination, limiting its sampling to small tissue volumes, allowing for difficultly distinguishable bladder wall changes like fibrosis to go unnoticed [47].

#### CT

A widely accredited indicator of pathological developments in bladder disease is bladder wall thickness (BWT) as it has been found to increase under inflammatory conditions [52]. In a large cohort prospective study, Jhang et al. found both focal and diffuse BWT – detected via Multiphasic CT urography – to be linked to the clinical manifestation of IC/BPS as well as to the histopathological findings consistent with ongoing inflammation: infiltration of pro-inflammatory cells, loss of uroepithelial cell lining, and synthesis of granulation tissue [53, 54]. CT mediated detection of BWT with its high sensitivity (95%) and specificity (92%) could, therefore, serve as proxy for detection of chronic bladder wall inflammation and fibrosis, therefore improving upon current diagnostic practices based on physicians’ personal judgement and on the adoption of radiological evidence solely to rule out alternative diagnoses [47, 53, 54].

#### US/MRI

**Fig. 4:**
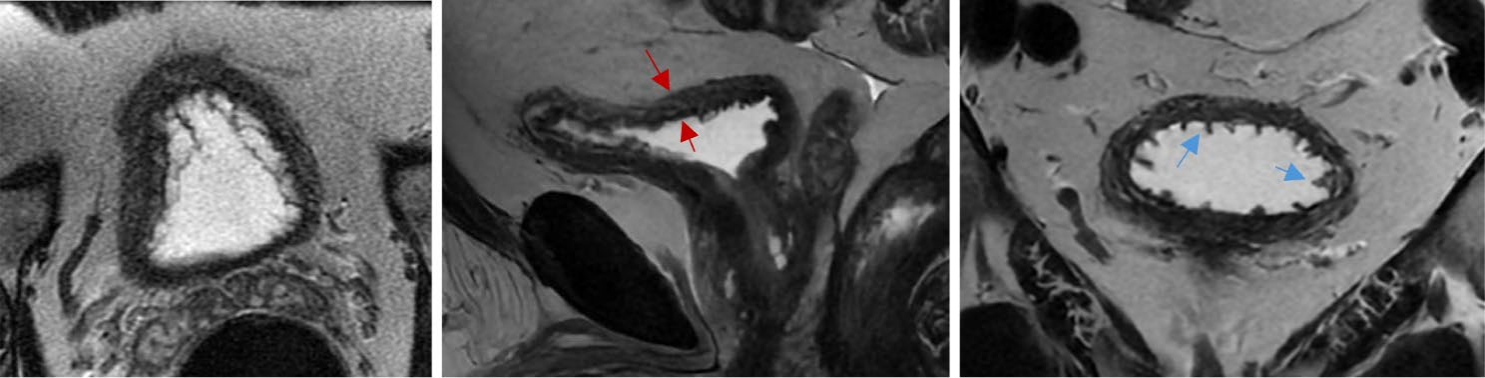
T1/T2 weighted MRI (axial, sagittal, and coronal T2 images) showing circumferential bladder wall thickening (red arrows) and trabeculation secondary to chronic outlet obstruction from benign prostatic hyperplasia (blue arrows)

Magnetic resonance urography and US are alternative imaging options for patients with contraindications to CT urography, such as pregnancy, contrast allergy, or renal insufficiency [53, 54]. MRI affords high contrast and spatial resolution of BWT, making it one of the preferred methods to produce three-dimensional images of the organ [47, 55]. Conventional MRI imaging is T1 and T2 weighted, relying on the differences in longitudinal and transverse relaxation times between neighboring tissues [55]. To increase image resolution and minimize artifacts, Tyagi et al. studied novel contrast mixtures (NCM) of gadolinium-based contrast agents and ferumoxytol [55]. NCM allowed for more accurate characterization of bladder wall boundaries with a 4-fold increase in contrast-to-noise ratio, a measure of image quality, thus holding promise for the future of noninvasive diagnoses of IC/BPS patients [55]. An example of T1 and T2 weighted MRI is provided in Fig. 4. It shows circumferential bladder wall thickening and trabeculation secondary to chronic outlet obstruction from concurrent benign prostatic hyperplasia. Finally, US may be considered in addition to CT urography, however, its use is not recommended on its own because its low sensitivity (50%)^54^.

### Future directions

**Table 2:** Bladder Fibrosis Imaging – Pros/Cons with respects to the gold standard

Lung Fibrosis Imaging	Cytoscopy^1^	PROs	Direct, Allows for biopsy, Real time, No ionizing radiation
		CONs	Operator dependent, Invasive, Small field of view, Only gross morphological discrimination
	Multiphasic CT Urography	PROs	Findings found to be linked to clinical manifestations and histopathology of IC/BPS, High sensitivity/specificity, Low acquisition time, Readily available
		CONs	Use of ionizing radiation
	MRI^2^	PROs	High contrast, High spatial resolution, Non-invasive, No ionizing radiation, Readily available
		CONs	Use of contrast agents, High cost
	US	PROs	Low-cost, Readily available, Well tolerated, No ionizing radiation
		CONs	Low sensitivity unless paired with CT, Operator dependent, Dependent on patient habitus

^1^Gold standard, ^2^Promising future techniques

Benefits and drawbacks of each imaging technique discussed above are summarized in Table 2. Among the proposed alternatives, the authors of this review feel T1/T2 weighted MRI to be the most promising (Fig. 4). Indeed, MRI affords high contrast and spatial resolution imaging of BWT (a widely accredited indicator of pathological developments in bladder disease), making it one of the preferred methods to produce three-dimensional images of the organ [47, 55]. Furthermore, it is ionizing radiation free, thus serving as a valid alternative for patients with contraindications to these [53, 54]. Finally, to further increase image resolution, minimize artifacts, and more accurately characterize bladder wall boundaries with a 4-fold increase in contrast-to-noise ratio, a novel contrast mixture consisting of gadolinium-based contrast agents and ferumoxytol can be employed [55].

## Prostate fibrosis

### Mechanism of injury

Benign prostatic hyperplasia (BPH) can be defined as the progressive enlargement of the prostate gland following the proliferation of cells constituting the periurethral tissue [56]. It is the most common benign neoplasm affecting millions of American males over the age of 30 and increases in prevalence with age [56–58]. BPH is detectable in nearly 40% of males during their 4th decade of life and then in nearly 90% of males in their 9th decade of life [56, 58–60]. BPH, however, is a purely histological definition and must be distinguished from the symptoms that may be secondary to it and which are referred to as lower urinary tract symptoms (LUTS) which include storage, voiding, and post-void symptoms [59–63]. Ultimately, if left untreated, these may progress to bladder dysfunction, urinary retention, and renal failure, severely impacting the patient’s quality of life [62–64]. To date, the cellular and molecular processes underlying the development of BPH and leading to LUTS are incompletely understood [59–61, 64]. Steroid hormones which are essential to normal prostate physiology have been found to play a key role in the disorder’s progression [57, 58, 60, 61, 64]. Androgen receptors are expressed in BPH tissue where potent dihydrotestosterone androgens activate them [60]. Hormonal factors alone do not exhaustively explain BPH development [64]. Accumulating evidence suggests the presence of additional and alternative etiologies including the effects of the sympathetic nervous system, varying levels of Insulin-like Growth Factor (IGF), genetic predispositions, physiological aging processes, infective processes, and, most importantly, systemic inflammation [58, 60, 61, 65]. BPH may be viewed as a form of chronic inflammatory prostatitis, whose pathogenesis may be triggered by a multitude of factors and pathways [61]. Tissue damage resulting from the above cited pathogenic pathways triggers the release of proinflammatory cytokines, chemokines, and growth factors, leading to local inflammation and prostate enlargement by means of epithelial and stromal cell proliferation [60, 61, 64]. This response is perpetuated by the release of prostatic self-antigens which sensitize the immune system and give rise to an autoimmune response [60, 61]. Following this chronic inflammation, periurethral prostate tissue undergoes the aberrant wound healing process of fibrosis with consequent prostate tissue stiffening [63, 65]. The gland’s ability to bend and expand to accommodate for urinary flow may be altered and could manifest as LUTS [63]. Gharaee-Kermani et al. have demonstrated the presence of a strong association between fibrotic changes in peri-urethral prostate tissue and the severity of LUTS in males [62].

Prostatic fibrosis appears dark on T2 weighted pelvic MRI [66]. It shows enhancement post-contrast and tends to be well defined with a rounded encapsulated appearance [67]. Prostatic pathologies can masquerade as prostate cancer; ADC, a measure of water diffusion within the tissue, is commonly calculated using DWI with the aim of differentiating the two types of prostatic lesion [67]. Finally, when MRI is not available, transabdominal and transrectal US (TRUS) offer an accessible and affordable alternative which is widely adopted [66].

### US

TRUS guided biopsy has been the gold standard prostate cancer diagnostic tool for decades, due to familiarity among physicians, ease of use, widespread availability, and affordability [66, 68, 69]. Although TRUS by itself has been found to be unreliable in the detection of cancerous lesions, with sensitivity and specificity ranging from 40 to 50%, it is superior to the highly subjective results of the digital rectal examination and has been found to be an effective tool for the measurement of prostate volume and the assessment of prostate anatoma [69–71]. Even so, the true advantage offered by TRUS in the realm of prostate density detection is in conjunction with bioptic technology. Indeed, by placing a small, lubricated US probe into the rectum, physicians can help themselves in properly orienting the biopsy needle’s trajectory with the aim of safely and effectively sampling the peripheral zone, where most cancers arise [69, 72]. Traditionally, TRUS guided biopsies obtain two cores per prostate sextant [72]. In so doing, it is possible that small, peripheral tumors, as well as transitional, central, or fibromuscular cancers may be missed despite their concerning potential for being aggressive and clinically significant (high false negative results varying from 17 to 21%)^68,70–72^. Similarly, non-significant peripheral tumors may be detected, resulting in over-diagnosis and over-treatment of low-grade indolent cancers estimated from 27 to 56%^68,70–72^. For all these reasons, while the use of TRUS in prostatic disease will most likely not dissipate anytime soon, other techniques, capable of curtailing these shortcomings should be further investigated [69].

An additional method for prostate examination is provided by USE [70, 71]. USE considers variations in soft tissue stiffness resulting from pathophysiological processes such as fibrosis and cancerous proliferation to single out affected tissue: the lower the estimated strain rate, the stiffer the tissue [70, 71]. This technology provides greater sensitivity for detecting prostate cancer and exhibits a high negative predictive value, ensuring that fewer cancers are missed in the peripheral zone of the prostate and reducing the number of necessary biopsies [70, 71]. It does so while remaining an inexpensive, versatile, and widely available bedside imaging modality [70]. Two USE subvariants have been developed: SE and SWE [71]. SE of the prostate is based on the analysis of tissue deformation subsequent to manually induced dynamic mechanical stress of the prostate tissue via the transrectal transducer probe itself [70, 71]. Tissue stiffness is thus estimated by visualizing the differences in strain between adjacent regions: hypoechoic hard lesions are highly suspicious for malignancy [71]. It is, however, unlikely that physicians be able to reliably maintain uniform compression over the entire prostate gland, introducing an intrinsic risk for operator dependent variability into tissue stiffness measurements [70]. To curtail this limitation, a water-filled balloon may be interposed between the probe and the rectal wall to improve the homogeneity of the deformation [71]. SWE, instead, requires no operator dependent compression of the rectal wall [71]. Indeed, while being maintained in a steady-state position, the endorectal transducer is capable of remotely inducing a US shear wave whose propagation velocity through the tissue is measured and related to prostate stiffness [71]. This technique provides quantitative measurement of elasticity values for each region of interest (ROI), resulting in the real-time realization of an elastogram: a color map of soft tissues elastic properties [70, 71].

### MRI

**Fig. 5:**
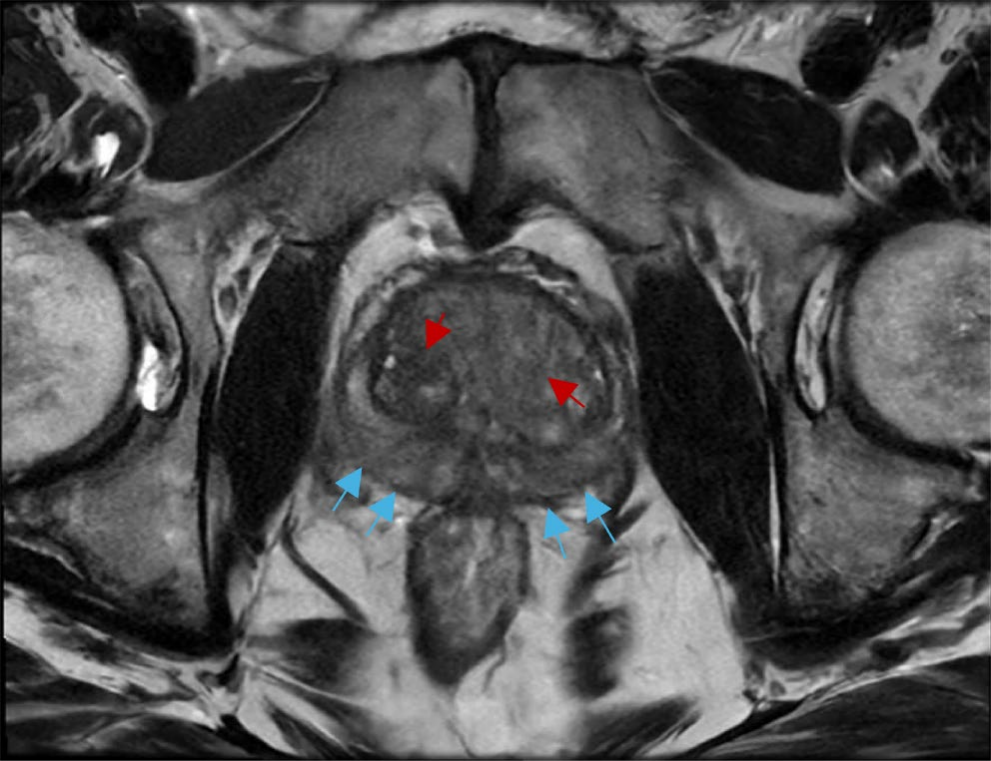
mpMRI of the prostate (Axial T2). Multinodular enlargement of the transition zone with diffuse heterogenous T2 hypointensity in the presence of BPH (red arrows). Symmetric diffuse enlargement and T2 hypointense scarring of the peripheral zone corresponding with sequela of chronic prostatitis (blue arrows)

**Fig. 6:**
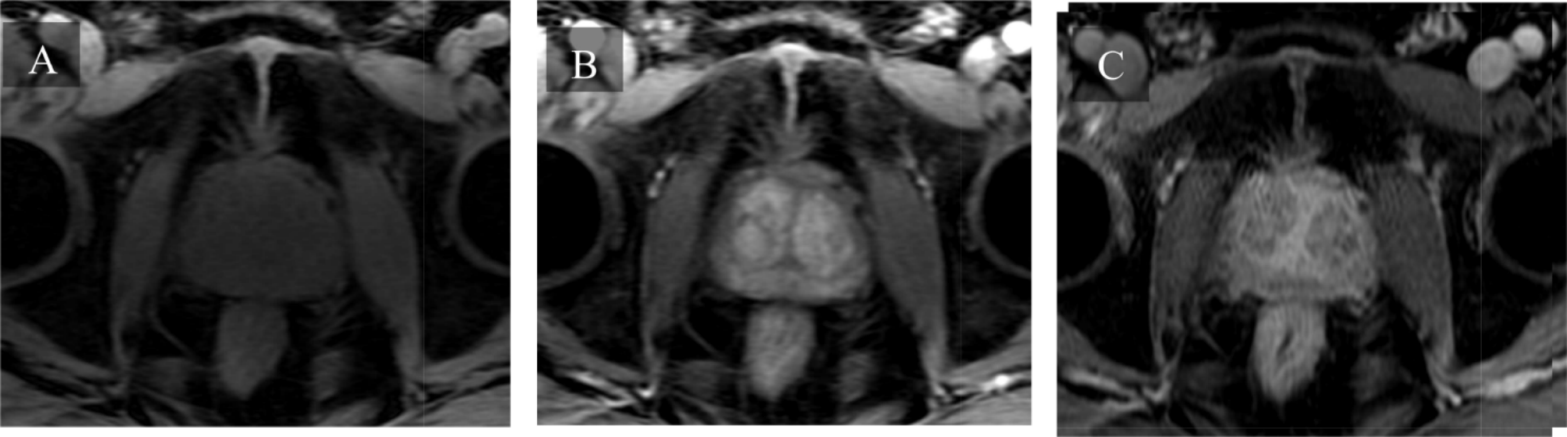
mpMRI of the prostate. Axial T1 pre-contrast arterial and delayed phase showing diffuse nodular enlargement

**Fig. 7:**
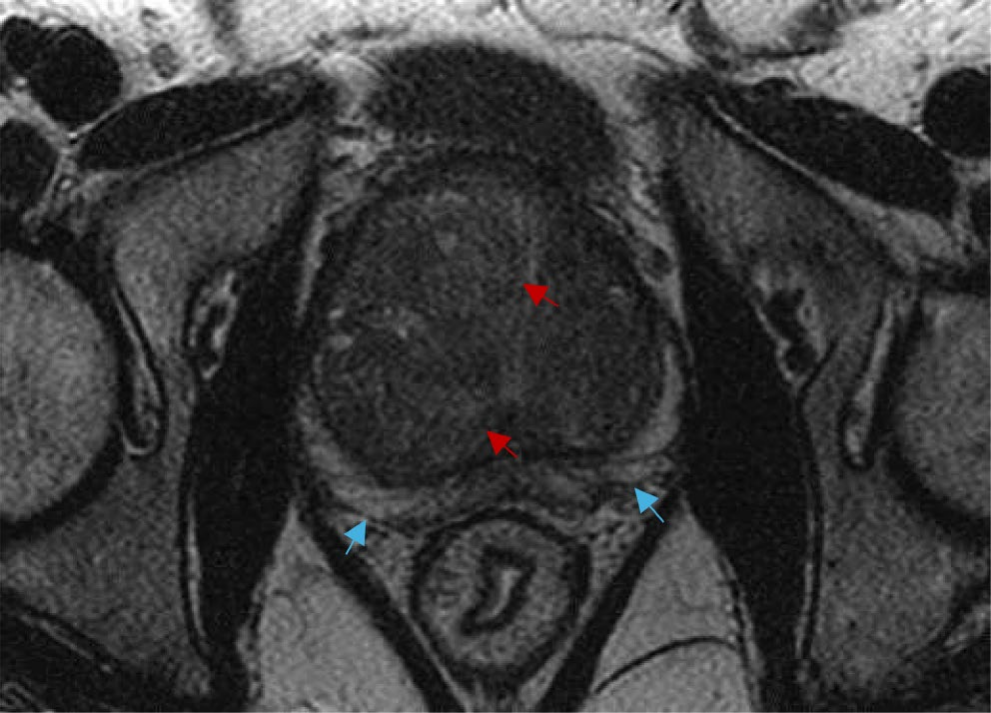
mpMRI of the prostate (Axial T2). Multinodular enlargement of the transition zone (red arrows) with diffuse heterogeneous T2 hypointensity corresponding with BPH. Notice the striated T2 hypointense scarring of the peripheral zone (blue arrows). BPH: benign prostatic hypeorplasia

**Fig. 8:**
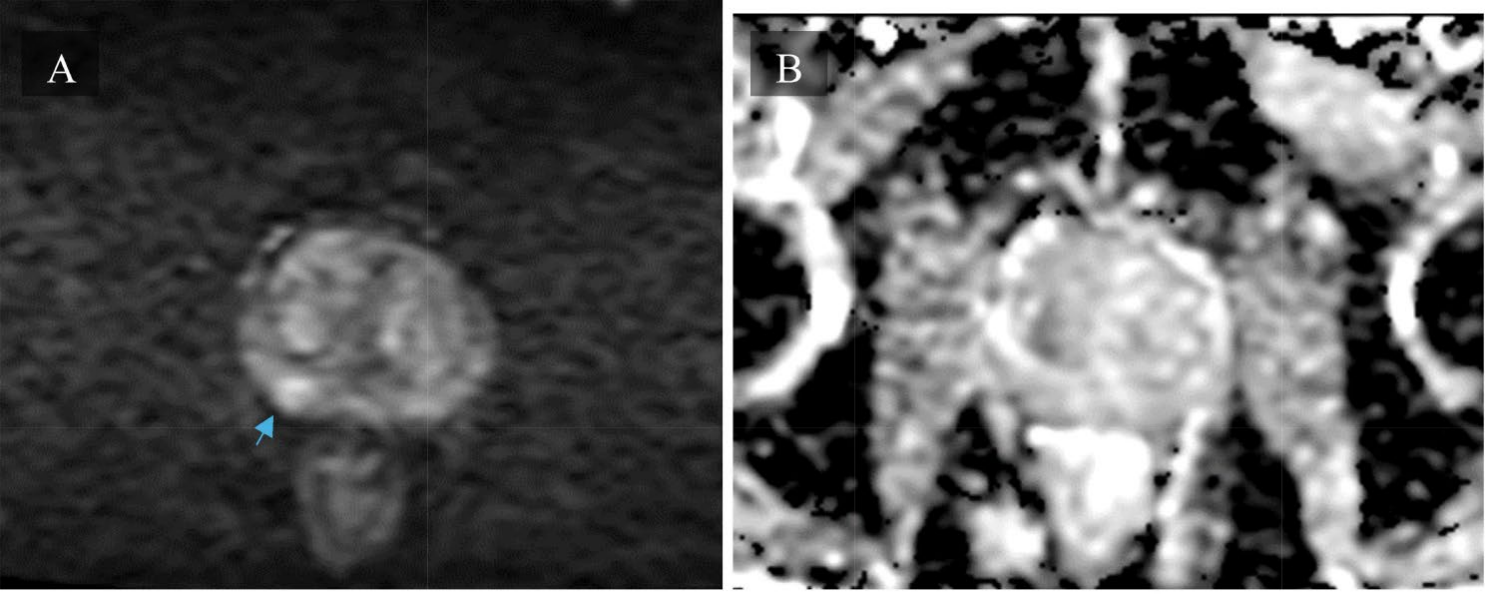
Axial DWI (**A**) & ADC (**B**) showing heterogenous nodular restricted diffusion throughout transition zone as well as low grade mass in right peripheral zone (blue arrow). DWI: Diffusion-weighted imaging. ADC: Apparent Diffusion Coefficient

An alternative tool for the diagnosis and characterization of prostate fibrosis is provided by multi-parametric MRI (mpMRI) [68]. Screening strategies involving the use of mpMRI rather than TRUS biopsies have shown higher sensitivity and specificity for both detection and localization of prostate cancer and fibrosis avoiding unnecessary repeated biopsies and reducing overtreatment [68, 72]. mpMRI makes use of three sequence modalities: one morphological sequence - T2 weighted imaging (T2W) - fused with two functional sequences - DWI and dynamic contrast-enhanced (DCE) images [68, 69, 72, 73]. Axial, coronal, and sagittal high-resolution T2W anatomical images assess the presence of structural abnormalities in the prostate and surrounding organs [68, 72, 73]. Some tumors, however, may appear isointense to the physiological prostate tissue, thus limiting diagnostic accuracy of isolated T2W imaging, leading to the need for DWI and DCE conjunction [68] (Fig. 5). DWI assesses cell density and subsequent variations in water diffusion rate within the prostatic interstitial space through the estimation of the ADC [67–69]. A decrease in DWI may be taken as a proxy for increased stromal density (i.e., BPH or neoplastic growth) [68]. Figure 5 provides an example of an axial DWI MRI showing heterogenous nodular restricted diffusion throughout the transition zone as well as a low grade mass in the right peripheral zone. Instead, DCE assesses variations in microvascular properties, angiogenesis and resulting perfusion rate, characteristic of histological variations, by way of gadolinium contrast agent [68, 69]. Through all these MRI modes of observation, prostatic fibrosis appears as wedge-shaped or band-shaped areas of dark hypo-intensity compared to the high signal intensity characterizing normal prostatic tissue [66, 68]. mpMRI imaging is standardized using the Prostate Imaging-Reporting and Data System (PI-RADS), which provides assessment criteria to rate the likelihood of PC being present on a scale from 1 to 5^69^. Figures 6 and 7, and 8 provide examples of mpMRI. The first shows multinodular enlargement of the transition zone with diffuse heterogenous T2 hypointensity in the concurrent presence of BPH and symmetric diffuse enlargement and T2 hypointense scarring of the peripheral zone corresponding to sequelae of chronic prostatitis. The second shows axial T1 pre-contrast arterial and delayed phase mpMRIs with diffuse nodular enlargement. The third shows multinodular enlargement of the transition zone with diffuse heterogeneous T2 hypointensity corresponding with BPH. Notice the striated T2 hypointense scarring of the peripheral zone.

### Nuclear medicine

The use of PET/CT, combining functional and morphologic information, for prostatic fibrosis and prostatic cancer (PC) imaging has been increasing within the last decade leading to fervent debate over the best contrast agents for the job [74]. Despite its widespread use in oncologic imaging, 18 F-FDG radiotracer does not play as important a role in the PET/CT imaging of PC because only highly aggressive, poorly differentiated, or undifferentiated PC has been shown to have a high glycolytic rate, thus limiting its sensitivity for localized and early metastatic disease [74, 75]. Among the most used PET/CT tracers for PC imaging in Europe are radiolabeled choline derivatives (18 F-fluorocholine or 11 C-choline) whose function is based on the increased uptake and turnover of phosphatidylcholin, an essential part of the phospholipids in the cellular membrane, seen in cancer cells [74, 75]. Diagnosis and primary staging of PC through such radiotracers are limited by their nonspecific uptake into benign intraprostatic tissues leading to relatively low sensitivity [74, 75]. For this reason, 18 F-fluorocholine or 11 C-choline have found most frequent application in restaging in the setting of biochemical recurrence, made evident through a rise in PSA levels following primary therapy of prostate cancer [74–76]. Recently, however, their application in this context has been replaced by that of prostate-specific membrane antigen (PSMA) which has shown higher diagnostic efficacy [65, 74–76]. Being highly overexpressed in PC cells while contemporaneously presenting low concentration in the bloodstream, PSMA, a type II transmembrane glycoprotein, sees an increase in its expression as tumor stage and grade heightens, rendering it an ideal target for high quality imaging and treatment [74–76]. Currently, the most widely used PSMA tracer is Gallium Ga 68 PSMA-11 (68Ga-PSMA-11) [75]. Compared to PSMA-PET/CT, standard-of-care imaging (CT, MRI, etc.) was found to have lower sensitivity (38% vs. 85%), specificity (91% vs. 98%), and precision (59–74% vs. 91–95%). For all these reasons, PSMA ligand PET/CT has quickly become a clinically accepted technique for recurrent PC imaging worldwide [74, 75].

68Ga-PSMA-11 radiotracer has found valid application also in PET/MRI for primary PC detection [75]. Indeed, when compared to PSMA enriched PET/CT, this technology presents with improved lesion detection, higher soft tissue contrast, and lower radiation dose to the patient [75]. The overall discrepancy in positive findings between PET/CT and PET/MRI has been found to be very low, with agreement ranging from 71 to 95%^77^. Instead, when compared to mpMRI, integrated PSMA PET/MRI has shown clear superiority in both staging and restaging. Indeed, it has proven to be of greater diagnostic value for the detection of cancers that are commonly missed on mpMRI having increased lesion contrast, excellent consistency in lesion detection, and higher sensitivity in the identification of primary tumors in the peripheral zone of prostate gland (74% vs. 50%; P, 0.001) [75, 77].

### Future directions

**Table 3:** Prostate fibrosis imaging– Pros/Cons with respects to the gold standard

Lung Fibrosis Imaging	TRUS Guided Biopsy^1^	PROs	Easy to use, Widespread availability, Low-cost, No ionizing radiation
		CONs	Low sensitivity/specificity, Invasive, High false negative rate due to sampling error, Over-diagnosis/treatment of benign peripheral tumours, Operator dependent, Dependent on patient habitus
	USE-SE/SWE	PROs	High sensitivity, High negative predictive value, Low-cost, availability, Quantitative, Real time, Readily available, Well tolerated, No ionizing radiation
		CONs	Operator dependant, Dependent on patient habitus
	mpMRI^2^	PROs	High sensitivity/specificity, Standardized reporting via PI-RADS, No ionizing radiation
		CONs	Low diagnostic accuracy, High cost
	18 F-FDG-PET-CT	PROs	Low sensitivity
		CONs	Use of contrast agent, Use of ionizing radiation, High acquisition time, Low availability, High cost
	PSMA-PET-CT	PROs	High sensitivity/specificity/precision
		CONs	Use of contrast agent, Use of ionizing radiation, High acquisition time, Low availability, High cost
	PSMA-PET-MRI	PROs	High soft tissue contrast, Low radiation dose, High sensitivity, No ionizing radiation
		CONs	Use of contrast agent, High acquisition time, High cost

^1^Gold standard, ^2^Promising future techniques

Benefits and drawbacks of each imaging technique discussed above are summarized in Table 3. Among the proposed alternatives, the authors of this review feel mpMRI to be the most promising. mpMRI has shown higher sensitivity and specificity for both detection and localization of prostate cancer and fibrosis avoiding unnecessary repeated biopsies and reducing overtreatment in low-risk cancers [68, 72]. mpMRI makes use of three sequence modalities, both morphological (T2W) and functional (DWI and DCE) [68, 69, 72, 73]. High-resolution T2W anatomical images assess the presence of structural abnormalities [68, 69, 72]. DWI images estimate ADC to assess cell density and subsequent variations in water diffusion rate [67–69]. Instead, DCE makes use of gadolinium contrast to assess variations in the microvasculature and the resulting perfusion rate [68, 69].

**Table 4:** Authors’ opinion about the most promising radiology techniques to diagnose fibrosis in each organ

Suspected affected organ	Promising radiology techniques for diagnosis
Kidneys	ASL MRE
Bladder	T1/T2 weighted MRI
Prostate	mpMRI

## Conclusions

Fibrosis is a pathological process characterized by abnormal deposition of connective tissue and improper tissue repair in response to sustained injury [1]. It can impact any organ, leading to severe structural and functional dysfunction and even failure [2, 3]. Aberrant tissue repair determines the development of chronic inflammation, excessive fibroblast proliferation, heightened collagen deposition, and, ultimately, an imbalanced alternation of scar formation and remodeling [3, 5]. While extensive research has already been carried out on the topics of aberrant wound healing and fibrogenesis, we lack a thorough understanding of how their relationship reveals itself through modern imaging techniques. Considering the profound implications that advancements in this field may carry, and with the objective of exploring and expanding upon our current understanding, this study sought to study fibrosis across various organs of the genitourinary system and catalog the foremost imaging technologies utilized for its identification. A comprehensive literature review has identified US, CT, MR, and PET as the most widely utilized imaging technologies for detecting fibrosis in organs of the genito-urinary system. Indeed, these are generally considered standard of care techniques, topped only by tissue specific approaches like cystoscopy for bladder fibrosis and elastography, an emerging technology, only recently gaining traction in routine clinical practice. Among the proposed alternatives, the authors of this review find MRI to be the most promising due to its superior soft tissue contrast, absence of ionizing radiation, and compatibility with elastography, DWI, and nuclear spin technology, among others. Additionally, MRI is widely available, permits full-body scanning, and has been reported to cause fewer allergic reactions compared to other contrast-exploiting techniques like X-ray and CT.

### Electronic supplementary material

Below is the link to the electronic supplementary material.

Supplementary Material 1

Supplementary Material 2

## Data Availability

Data sharing not applicable to this article as no datasets were generated or analyzed during the current study.

## Abbreviations

FDG: 18 F-Fluorodeoxyglucose
AE-IPF: Acute Exacerbation of IPF
ABUS: Automated Whole-Breast Us
BBD: Benign Breast Disease
BCT: Breast Computed Tomography
BI-RADS: Breast Imaging Reporting And Data System
BAL: Bronchoalveolar
CMR: Cardiac Magnetic Resonance
CEMDCT: Contrast Enhanced Multi-Detector CT
CEBCT: Contrast-Enhanced Breast CT
DBT: Digital Breast Tomosynthesis
TE: Echo Time
ECV: Extracellular Volume
He: Helium
HRCT: High Resolution Computed Tomography
IPF: Idiopathic Pulmonary Fibrosis
IGF-I: Insulin-Like Growth Factor I
IGFBP-3: Insulin-Like Growth Factor-Binding Protein 3
IL-8: Interleukin-8
LGE: Late Gadolinium Enhancement
M-CSF: Macrophage Colony-Stimulating Factor
MRI: Magnetic Resonance Imaging
MMPS: Matrix Metalloproteinases
MCP-1: Monocyte Chemotactic Protein-1
NK Cells: Natural Killer Cells
PDGF: Platelet-Derived Growth Factor
PET: Positron Emission Tomography
PAR: Protease Activated Receptors
Q-CT: Quantitative CT
ROS: Reactive Oxygen Species
RAAS: Renin-Angiotensin-Aldosterone System
STE: Speckle Tracking Echocardiography
TIMPS: Tissue Inhibitors Of Metalloproteinases
TGF-Β1: Transforming Growth Factor Β1
US: Ultrasound
UTE: Ultrashort Echo Time
Xe: Xenon
ZTE: Zero Echo Time
