# Supplementary material for: Radiology of fibrosis part III: genitourinary system
Source: J Transl Med. 2024 Jul 3;22:616. doi: 10.1186/s12967-024-05333-1 (PMC11223291; doi:10.1186/s12967-024-05333-1)
Supplement: Supplementary file 2 — Supplementary Material 2 [file 12967_2024_5333_MOESM2_ESM.docx]

To Whom It May Concern:

I am writing to resubmit our manuscript titled "Radiology of Fibrosis Part I: Thoracic Organs" for consideration in The Journal of Translational Medicine, following the revisions suggested by the esteemed reviewers. We are immensely grateful for the insightful feedback provided during the initial review process, and we believe that the revisions we have made substantially strengthen the manuscript.

Please find bellow a comprehensive list of changes made to the original manuscript in response to each Reviewer’s suggestions.

**Reviewer #1**

- “The figures need to include the details of the imaging sequences (i.e. MRI, T1/2/Flair/Fat suppression, TR/TE, etc.)…”
  - Thank you for taking the time to revise our work. We agree with your suggestion and have thus ensured the details of each imaging sequence are included in their description.
- “…distinguishing characteristics that need to be included in tabular format”
  - We appreciate your thoughts and have created 3 new tables in which the charachteristics of each imaging technology are displayed in a clear and concice manner.
- “The most radiology genomics correlation maybe considered”
  - We sincerely appreciate your insightful comments. However, after careful consideration, we have chosen not to implement your advice, as we believe it falls outside the scope of our paper.
- “A figure and schematic could also result in improvement” and “The two tables need to include more details”
  - We appreciate your insightful evaluation of our work and concur that integrating additional schematics/figures and enriching the tables with more comprehensive data could enhance the quality of our manuscript. As a response, we have included a detailed schematic elucidating the pathophysiology of fibrosis, which will be seamlessly integrated into the inaugural segment of our trilogy. While this specific schematic is not appended to this current paper, it aligns with the recommendation from one of your esteemed colleagues to streamline the background section, directing readers to the initial part of our trilogy for a deeper exploration of fibrosis. Moreover, we have revamped Table 1 into a set of three new tables—Tables 1, 2, and 3. These tables comprehensively outline the merits and demerits of all referenced imaging modalities for each relevant organ. They also highlight the current gold standard alongside what we perceive as the most promising technology for future fibrosis imaging endeavors. This strategic arrangement enables readers to make direct comparisons between the two, facilitating a more informed understanding of the landscape.
- Also, the text has some usage of common words (colloquial) and needs to be grammatically checked
  - Thank you for taking the time to review our paper. We have taken your thoughts into consideration and made changes to the wording of our work.

**Reviewer #2**

1. For Figures 1 through 8, the author provides detailed textual descriptions. Could the author please consider adding arrows to these descriptions to help readers better understand what fibrosis looks like on imaging?
   1. Thank you for dedicating your time to reviewing our work. We acknowledge and appreciate your feedback regarding the need to cite the figures within the main body of the text. We have taken this suggestion into account and ensured that the figures are appropriately cited within the main body. Furthermore, arrows have been added to each image with the aim of facilitating their interpretation.
2. Regarding Table 1, could a brief sentence summarizing the radiological manifestations of fibrosis for each organ be included, allowing readers to learn more intuitively?
   1. We appreciate your insightful evaluation of our work and concur that enriching the tables with more comprehensive information could enhance the quality of our manuscript. As a response, we have revamped Table 1 into a set of three new tables—Tables 1, 2, and 3. These tables comprehensively outline the merits and demerits of all referenced imaging modalities for each relevant organ. They also highlight the current gold standard alongside what we perceive as the most promising technology for future fibrosis imaging endeavors. This strategic arrangement enables readers to make direct comparisons between the two, facilitating a more informed understanding of the landscape.
3. The author has provided descriptions of typical manifestations of fibrosis in various organs. Could additional descriptions of differential diagnoses for diseases with similar clinical and radiological manifestations to those in the urinary system be added?
   1. We sincerely appreciate your insightful comments. However, after careful consideration, we have chosen not to implement your advice, as we believe it falls outside the scope of our paper.

**Reviewer #3**

1. Just would like to see the Part I , II and III abstract and background are not repetitive and has a connect and flow
   1. Thank you for your insightful feedback. We value your input and have taken steps to enhance the coherence and flow of the text across the three parts of this series. Specifically, we have addressed repetition and improved the connectivity between sections. To achieve this, we have omitted the detailed, step-by-step description of fibrosis pathophysiology from the paper's background. Additionally, we have introduced a new paragraph elucidating the tripartite nature of our work.
2. The figure numbers need to be referred in text and individual figures need to be explained with arrows where you detect changes in fibrosis using the mentioned technique
   1. Thank you for dedicating your time to reviewing our work. We acknowledge and appreciate your feedback regarding the need to cite the figures within the main body of the text. We have taken this suggestion into account and ensured that the figures are appropriately cited within the main body. Furthermore, arrows have been added to each image with the aim of facilitating their interpretation.
3. Overall conclusion needs more detailed tabulation of techniques in seen scenarios against each organs and where it stands out as treatment regimen
   1. We appreciate your insightful evaluation of our work and concur that enriching the tables with more comprehensive information could enhance the quality of our manuscript. As a response, we have revamped Table 1 into a set of three new tables—Tables 1, 2, and 3. These tables comprehensively outline the merits and demerits of all referenced imaging modalities for each relevant organ. They also highlight the current gold standard alongside what we perceive as the most promising technology for future fibrosis imaging endeavors. This strategic arrangement enables readers to make direct comparisons between the two, facilitating a more informed understanding of the landscape.
